# Relevant Assay to Study the Adhesion of *Plasmodium falciparum*-Infected Erythrocytes to the Placental Epithelium

**DOI:** 10.1371/journal.pone.0021126

**Published:** 2011-06-24

**Authors:** Philippe Boeuf, Wina Hasang, Eric Hanssen, Jocelyn D. Glazier, Stephen J. Rogerson

**Affiliations:** 1 Department of Medicine (RMH/WH), The University of Melbourne, Royal Melbourne Hospital, Melbourne, Australia; 2 Department of Biochemistry, LaTrobe University, and Electron Microscopy Unit, Bio21 Institute, The University of Melbourne, Melbourne, Australia; 3 Maternal and Fetal Health Research Centre, School of Medicine, Manchester Academic Health Sciences Centre, University of Manchester, St. Mary's Hospital, Manchester, United Kingdom; University of Copenhagen, Denmark

## Abstract

In placental malaria, *Plasmodium falciparum*-infected erythrocytes adhere to the apical plasma membrane of the placental epithelium, triggering an impairment of placental function detrimental to the fetus. The design of anti-adhesion intervention strategies requires a detailed understanding of the mechanisms involved. However, most adhesion assays lack *in vivo* relevance and are hardly quantitative. Here, we describe a flow cytometry-based adhesion assay that is fully relevant by using apical epithelial plasma membrane vesicles as the adhesion matrix, and being applicable to infected erythrocytes directly isolated from patients. Adhesion is measured both as the percentage of pathogens bound to epithelial membrane vesicles as well as the mean number of vesicles bound per infected erythrocytes. We show that adhesins alternative to those currently identified could be involved. This demonstrates the power of this assay to advance our understanding of epithelial adhesion of infected erythrocytes and in the design of intervention strategies.

## Introduction

Polarized epithelia line organs such as the stomach and intestines, lung, and placenta. They are the first line of defense against a variety of pathogens, many of which have developed mechanisms to adhere to epithelia to survive in their host. Pathogens can either adhere directly to the epithelium, or infect and modify host cells to cause adhesion. These pathogens are highly prevalent and range from viruses like the cytomegalovirus to bacteria like *Helicobacter pylori* or parasites such as the malaria agent *Plasmodium falciparum*. The health and economic impacts of these infections are immense.

Epithelial adhesion is the first step in the pathogenetic process of such pathogens. In some cases, adhesion may also allow a pathogen to evade clearance. Altered function of the epithelium may be followed by epithelial cell damage or destruction, and invasion into normally sterile sites such as the blood stream or the lung parenchyma. Decreased host fitness, and ultimately death, may occur.

Strategies aimed at preventing pathogen epithelial adhesion are needed and require a complete understanding of the underlying molecular mechanisms. This can only be achieved through adequate adhesion assays. However, assays currently available to address epithelial adhesion of pathogens have serious limitations. Most current assays use an adhesion matrix with no or limited relevance to the apical membrane of the epithelium studied. Some assays use cell lines (either non-polarized or artificially polarized) but these hardly model the host's epithelium [1] and expression profiling and functional studies of these cells suggest they often are quite different from the epithelium they intend to model [2]. Other assays make use of previously identified pathogen receptors adsorbed on plastic as an adhesion matrix and are obviously limited in their scope [3]. The receptors lack their *in vivo* molecular context; they may not be correctly folded and receptor synergy cannot be studied. As such, they may not support pathogen adhesion in the same manner or to the same extent as the native receptor [4]. Animal models also have limitations, as adhesion does not always occur at the same anatomical site as in the natural host [5] and the anatomical and histological characteristics of the target and human epithelia may differ [6].

Model pathogens are not always representative of those infecting the natural host, and some pathogens cannot be modeled, either because they cannot be maintained in culture or because they cannot infect laboratory animals (for example, *S. enterica* serotype Typhi can only infect humans [7]). Moreover, adaptation to culture conditions is associated with profound changes in the expression of virulence factors or adhesion phenotype in some pathogens [8], [9], [10].

Adhesion can quickly trigger a response from both the pathogen (invasion of the epithelium) and the epithelium (alteration of cell surface expression patterns) complicating the investigation of the early stages of adhesion in cellular or animal models [11]. An acellular assay would advance the dissection of the mechanisms underlying pathogen adhesion without interference from the epithelium response. New tools to prevent epithelial adhesion of pathogens are needed given the global health impact of these diseases. The successful design and validation of these anti-adhesion approaches requires an adhesion assay able to quantitatively measure adhesion and to easily and rapidly test the adhesion-blocking capacity of candidate therapeutics. However, most current assays are not quantitative and almost all are low throughput.

Here, we describe a novel assay that addresses these issues: it uses apical plasma membrane vesicles of the target epithelium as adhesion matrix and is applicable to pathogens directly isolated from infected hosts. Being an acellular approach using an isolated epithelial plasma membrane, it allows the investigation of the mechanisms of adhesion without interference from the epithelium response. The use of flow cytometry makes it quantitative, objective, reproducible and high throughput. Its use should both advance our basic knowledge of the mechanisms of epithelial adhesion as well as allow the design and validation of anti-adhesion strategies.

We applied this innovative adhesion assay to malaria-infected erythrocyte placental adhesion. After invasion of an erythrocyte, *P. falciparum* exports molecules to the surface of the infected cell. Some of these molecules support the adhesion of the infected erythrocyte to host tissues, including to the placental epithelium. Adhesion of *P. falciparum*-infected erythrocytes (IE) to the placenta can trigger local inflammation leading to placental impairment and fetal growth restriction [12]. A more complete understanding of the mechanisms involved in the placental adhesion of IE is required, so that strategies to prevent adhesion can be developed to improve maternal and fetal health. The mechanisms underlying the placental adhesion of IE are relatively well documented [12], offering a reliable way to validate our assay. Current understanding is that VAR2CSA, a parasite protein exported to the surface of the IE, binds to chondroitin sulfate A (CSA) expressed on the apical membrane of the placental syncytiotrophoblast epithelium, and high levels of antibodies against CSA-binding parasite lines are associated with better pregnancy outcomes [13]. However, research that has focused on the VAR2CSA/CSA interaction has largely ignored other potential parasite adhesins and placental receptors.

## Results

### Assay principle

**Figure 1:**
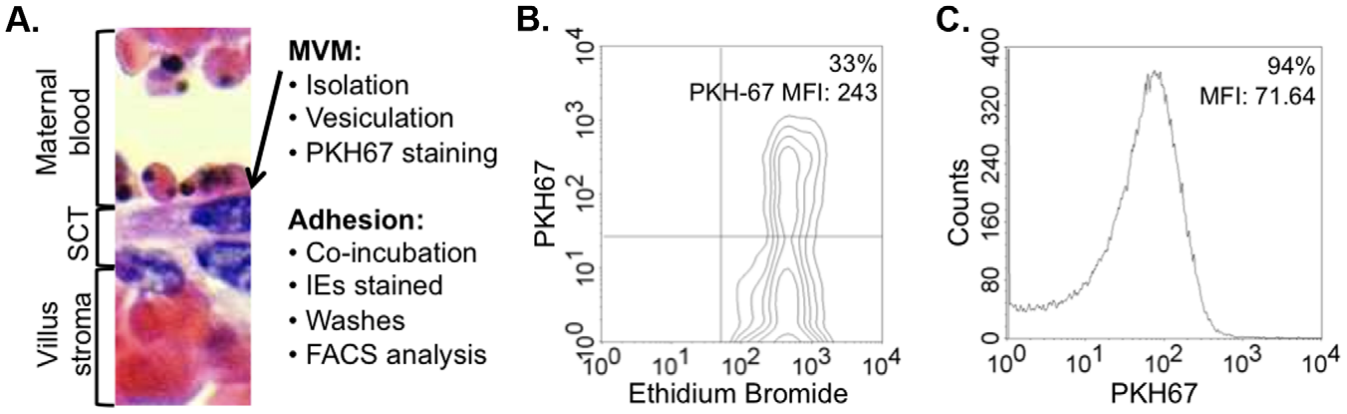
Assay principle. (**a.**) The epithelial microvillous plasma membrane (MVM) is purified from the host epithelium (the syncytiotrophoblast, or SCT, layer of the human placenta in this case) and forced to vesiculate. Vesicles labeled with the fluorescent lipid dye PKH-67 are co-incubated with pathogens (ethidium bromide-stained *P. falciparum*-infected erythrocytes (IE) of the placental-type line CS2 in this example). Unbound vesicles are washed off prior to acquisition on a flow cytometer. (**b.**) A typical cytogram shows that 33% of IE (ethidium bromide^+^ erythrocytes) have bound vesicles detected by their PKH67 fluorescence. The adhesion intensity is measured by the mean PKH67 fluorescence intensity (MFI) of pathogens bound to vesicles (243 in the example given). (**c.**) The PKH67 MFI of pathogens bound to vesicles is used to calculate the average number of vesicles bound per pathogen by dividing it by the PKH67 MFI of the vesicles used derived from the histogram shown. In the example given, each IE that adhered to vesicle was bound to an average of 243/71.64 = 3.4 vesicles.

Based on their highly negative surface charge density, the apical plasma membrane of several epithelia can be purified using a protocol based on Mg^2+^ precipitation and differential centrifugation [14]. Such plasma membrane fragments when isolated from human placenta and then forced to vesiculate by shear force, orientate right-side-out (i.e. the same orientation with respect to cytoplasm *in vivo*) [14]. The vesicles of the apical plasma membrane so generated (hereafter referred to as ‘vesicles’) are then labeled using a non-specific fluorescent lipid dye (PKH67 in this case) before being co-incubated with the pathogen of interest. Being non-specific, the lipid dye used is less likely to interfere with adhesion. If needed for identification, the pathogen can be fluorescently labeled (using ethidium bromide to stain its DNA for example). Vesicles that are not bound to pathogens are washed off before acquisition on a flow cytometer (Figure 1). Adhesion is quantified both as the percentage of pathogens bound to vesicles (percentage adhesion) as well as the mean PKH67 fluorescence intensity of vesicle-bound pathogens (adhesion intensity). A follow-up of the adhesion levels and intensity during flow cytometry acquisition reveals that the acquisition of as few as 950 pathogens (acquired in 8 seconds) are needed to obtain data statistically representative of the sample (data not shown).

The purity of the apical plasma membrane vesicle preparations was measured by determining the enrichment in alkaline phosphatase activity, a marker of the apical plasma membrane, as compared to the starting tissue homogenate [14]. The enrichment factors for the vesicles used in this study (median: 25.5; IQR: 22.91–34.07) accord well with previous values and indicate a high degree of purity.

### Assay validation: *Plasmodium falciparum*-infected erythrocytes adhesion to the placenta

We confirmed that our adhesion assay could recapitulate known features of the adhesion of IE to the placenta. By applying it to *var2csa*KO parasites, thereby abolishing the opportunity for VAR2CSA/CSA interaction, we investigated whether alternative adhesins could mediate placental adhesion.

#### Optimal vesicle/IE ratio

**Figure 2:**
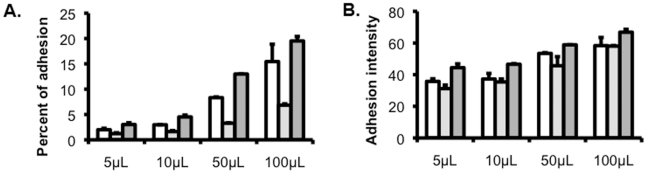
Adhesion level and intensity. Vesicles were prepared from three different placentas (denoted by the white, light grey and dark grey bars) and adjusted to the same density. Increasing volumes of these vesicles (5 to 100 µL) were incubated with the placental-type line CS2. (**a.**) Despite the similar purity and mean fluorescence levels of the various vesicle preparations, the percentage of IE with adherent vesicles varied significantly across vesicle preparations and increasing the volume of vesicles had a strong impact on the adhesion levels associated with some, but not all, vesicle preparations. (**b.**) This difference was not mirrored by differences in adhesion intensity (measured as the PKH67 MFI of vesicle bound-IE), which increased in proportion to vesicle volume for all vesicle preparations. Data are shown as mean and standard deviation from experimental triplicates.

In order to select the optimal volume of vesicle suspension to be used in comparative experiments, we measured adhesion as a function of increasing volume of vesicle suspension both as percentage adhesion (the percentage of IE with one or more PKH67-labeled vesicle bound) and adhesion intensity (PKH67 MFI of vesicle bound-IE) (Figure 2).

#### Number of vesicles per IE

**Figure 3:**
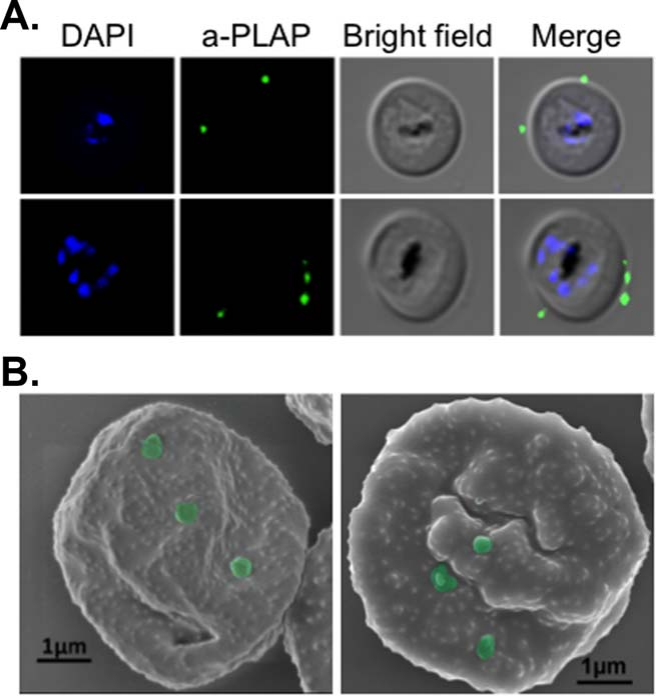
Adhesion intensity. (**a.**) Immunofluorescence with a vesicle-specific anti-placental alkaline phosphatase (PLAP) antibody and DAPI to stain the parasite DNA reveals a small number (2–4) of vesicles at the surface of IE. (**b.**) This is confirmed by scanning electron microscopy in which vesicles have been false-colored in green. Knob-like structures seen at the surface of the IE result from the extensive remodeling of the erythrocyte membrane by *Plasmodium falciparum* molecules. Bar size: 1 µm. Parasite line used: CS2.

The number of vesicles bound per IE was estimated by dividing the PKH67 MFI of vesicle-bound IE by that of vesicles alone, as described in Figure 1. Extrapolation of the data shown in Figure 2 predicted that the average number of vesicles/IE would start plateauing at 3.98±0.22. This is further supported by imaging of 2 to 4 vesicles at the surface of IE using either immunofluorescence (using an anti-placental alkaline phosphatase antibody) or scanning electron microscopy (Figure 3).

#### Variation in the ability of different parasite lines to bind to vesicles from different placentas

**Figure 4:**
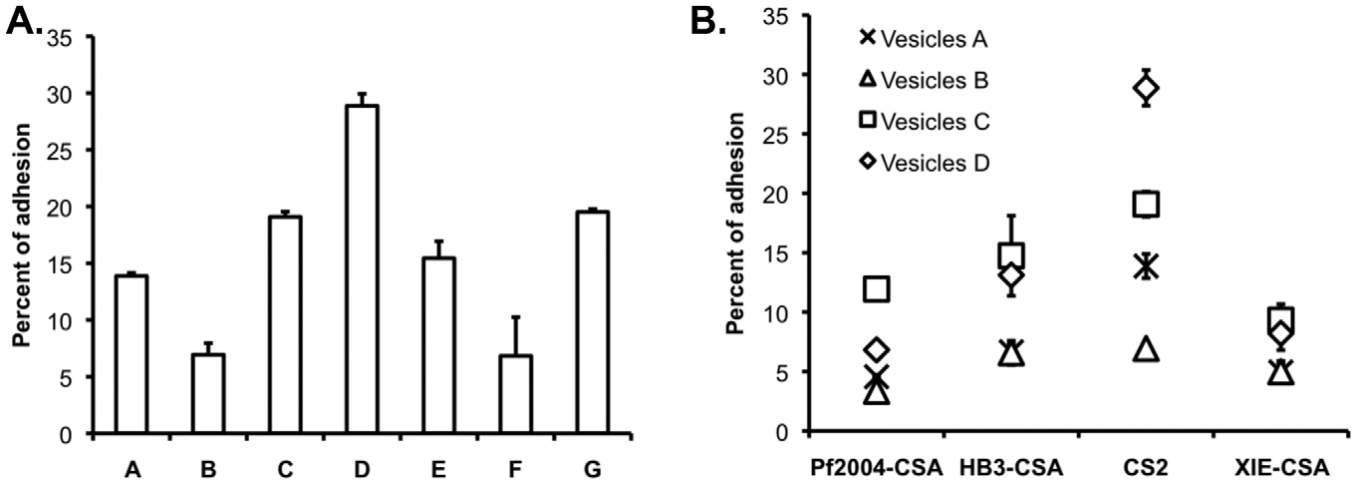
Inter-vesicle and inter-parasite line variations in adhesion. (**a.**) Vesicles were prepared from seven different placentas (A to G) and incubated with IE of the CSA-binding line CS2 at identical vesicle/IE ratios. The percentage of adhesion (percentage of IE with bound vesicles) shows a marked difference in the ability of preparations from different donors to bind to CS2 IE. (**b.**) Four vesicle preparations representative of the range of CS2 adhesion levels observed were selected (A to D) and adhesion levels of four different CSA-binding lines from various geographical origins were measured. A hierarchy exists amongst parasite lines as, regardless of the vesicle preparation used, CS2 shows the highest adhesion levels amongst all parasite lines tested. Such a hierarchy is not found across vesicle preparations as CS2 binds best to vesicles D, whereas other lines adhere better to vesicles C. Results are expressed as mean and standard deviation from experimental triplicates. Data representative of two independent experiments are shown.

Vesicles were prepared from placentas from seven different women, and their capacity to support adhesion of CSA-binding parasites was compared at similar vesicle/IE ratios (Figure 4a). Despite being of comparable density, purity and mean fluorescence intensity, these different vesicles showed significant variation in their capacity to support adhesion.

We used four different vesicle preparations (vesicles A to D from Figure 4a) to compare the binding levels of four placental-type parasite lines from different geographical origins [15]: Pf2004-CSA (West African), HB3-CSA (from Central America), CS2 (presumed from Brazil) and XIE-CSA (from Papua New Guinea). All parasite lines bound at high levels to CSA, and were tested at a parasitemia of 5% mature adhering trophozoite stages. When each parasite line was incubated with the same volume of the various vesicle preparations, we found a large variation in the ability of different lines to adhere to vesicles.

### Recapitulation of known features of *P. falciparum* placental adhesion

**Figure 5:**
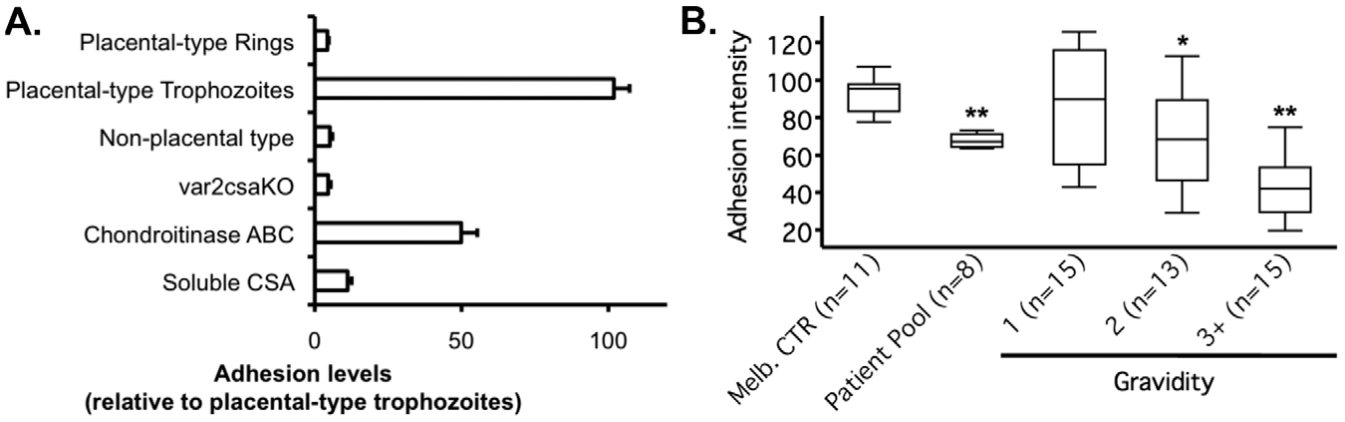
Recapitulation of known characteristics of malaria parasite placental adhesion. (**a.**) When normalized to the adhesion levels of VAR2CSA-expressing, CSA-binding placental-type trophozoites (CS2 line), earlier parasite ring stages show very limited binding. Trophozoites of the non-CSA binding parental line E8B (non-placental type) do not show significant adhesion to vesicles. The inhibition of adhesion of placental-type trophozoites by the disruption of *var2csa* (*var2csa*KO), treatment of vesicles with chondroitinase ABC or pre-incubation of IEs with soluble CSA suggests that most of the adhesion is VAR2CSA/CSA mediated. Data are shown as mean and standard deviation from experimental triplicates. (**b.**) The assay could also detect a parity-dependent adhesion inhibition by sera from malaria-exposed pregnant women. When normalized to the adhesion intensity in the absence of serum, sera from non-pregnant controls from Melbourne (Melb. CTR) did not induce any significant decrease in adhesion intensity whereas a pool of patients' sera selected for their high immunoglobulin titers against CS2 did. Sera from malaria-exposed pregnant women showed a parity-dependent increase in their adhesion blocking capacity. Boxes show the median, 25^th^ and 75^th^ percentiles and whiskers are 5^th^ and 95^th^ percentiles from all sera tested in triplicates. *: p = 0.046; **: p≤0.0003.

In order to validate our adhesion assay, we examined known characteristics of malaria parasite placental adhesion [12] (Figure 5).

After invasion of an erythrocyte, *P. falciparum* progresses from the ring stage that expresses few adhesins at its surface to the mature trophozoite stage at which the surface expression of adhesins is maximal. A culture of the CSA-binding CS2 line containing both ring and trophozoite stages was incubated with vesicles. As expected, the percentage of vesicle-binding ring stage IE was low (4.3%) when normalized to trophozoite stage IE, confirming the late stage specificity of IE adhesion to vesicles [3], [16].

Adhesion of IE to the placenta is specific for parasites infecting pregnant women [17]. This feature was recapitulated in our assay since the adhesion of the non CSA-binding E8B parasite line was only 5.1% of the level observed with CS2 IE.

We next demonstrated a major role for the VAR2CSA/CSA interaction in adhesion of IE to placental vesicles [18]. Cleaving CSA from vesicles with chondroitinase ABC inhibited the adhesion of placental-type IE by 50.1% (±5.5%), and pre-coating of IE with soluble CSA reduced binding to vesicles by 88.8% (±1.4%). The disruption of the *var2csa* gene in CS2 reduced adhesion by 95.5% (±0.9%) compared to parental CS2 IE (Figure 5a).

In malaria-endemic areas, women in their first pregnancy (primigravidae) are immunologically naïve to placental-type parasites, but a humoral immunity is developed through successful pregnancies and multigravidae have high levels of antibodies against placental-type parasites. One function of these antibodies is to block the adhesion of IE to the placenta. We compared the adhesion-inhibition capacity of serum samples from primigravidae, secundigravidae and multigravidae to sera from non-pregnant controls. As expected, the adhesion of the placental-type parasite line CS2 to vesicles was inhibited in a parity-dependent manner (Figure 5b).

### Assay application: aiming to identify alternative parasite adhesins involved in placental malaria

**Figure 6:**
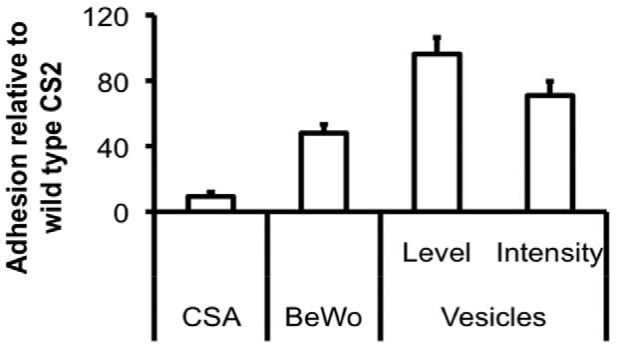
Adhesins other than VAR2CSA support adhesion to the placental epithelium. CS2 IE with disrupted *var2csa* were selected on BeWo cells for adhesion. In comparison to wild type CS2 IE, they bound at very low levels to isolated CSA. Their adhesion to the BeWo placental cell line was about half that of the wild type IE. However, in our vesicle adhesion assay, this BeWo-selected *var2csa*KO showed similar percentages of IE bound to vesicles (p = 0.7728) as well as comparable adhesion intensity (p = 0.2454) to wild type CS2. All results are normalised to wild type levels and represented as mean and standard deviation.

Our assay uses the actual membrane IE adhere to *in vivo*, so it is ideal to identify all potential parasite adhesins (and placental receptors) supporting IE placental adhesion. Since *var2csa* disruption did not totally suppress placental adhesion (Figure 5a), parasite adhesins other than VAR2CSA could mediate placental adhesion. We used *var2csa*KO IE repeatedly selected for their capacity to adhere to the placental cell line BeWo [19] as a model for a non-VAR2CSA expressing placental-type parasite line. The adhesion levels and intensity of this selected KO were compared to the wild type parasites (Figure 6). The selected KO showed reproducibly high levels of binding to vesicles (100%±2.63%) despite a 30% decrease in adhesion intensity compared to the wild type line. This suggests that parasite adhesins, other than VAR2CSA, can mediate very significant levels of placental adhesion. Their identification is crucial for the design of a fully protective vaccine against placental malaria.

## Discussion

Adhesion to the apical membrane of polarized epithelia is a key pathogenetic process of a variety of pathogens, including the malaria parasite *P. falciparum*. Studying the mechanisms underlying adhesion with a relevant, high throughput and quantitative assay would allow a better understanding of those mechanisms. Ultimately, this could also help in the design and validation of adhesion-blocking strategies aimed at improving health outcomes.

### Superiority over other assays

Most available adhesion assays lack relevance, either because of the adhesion matrix or the model pathogen they use. Because of these limitations, data generated from these assays must be treated with caution. As shown in Figure 6, model cell lines or, *a fortiori* isolated receptors, may not recapitulate the complex molecular profile of the apical epithelial membrane as well as vesicles can. By using the actual plasma membrane that a pathogen adheres to *in vivo*, and by being applicable to pathogens directly isolated from their host, our innovative adhesion assay brings a high level of relevance to the fundamental research into the mechanisms underlying epithelial adhesion of pathogens. In parallel, the use of flow cytometry allows the rapid and objective quantification of adhesion and opens avenues of research into the design and validation of adhesion-blocking strategies through drug discovery or vaccine design. To our knowledge, our adhesion assay is the only one to integrate adhesion matrix and pathogen relevance as well as high-throughput.

### Adaptability of the approach to other polarized epithelia and pathogens

We chose to validate our approach by applying the assay to the adhesion of malaria-infected cells to the placenta as the bases for this adhesion are well documented [12] and this pathogen can be cultured *in vitro*. However, the Mg^2+^ precipitation and differential centrifugation approach used to purify the apical membrane of the placental epithelium has been successfully applied to other epithelia with little or no modification [20], [21], [22]. The low number of pathogens required in the assay, allows its application to field isolates prior to their adaptation to culture. Finally, the quantity of starting material needed to purify apical epithelial vesicles is small and 5 assays can be performed on vesicles purified from only 10 g placental villous tissue (about 3% of the total weight of the human placenta). We are therefore confident that the approach we describe can successfully be transferred to other epithelia which have a microvillous surface with a highly negative surface charge density (such as intestine) and to other pathogens provided the pathogens could be isolated or cultured and fluorescently labeled, allowing detection by flow cytometry.

### Limitations of our assay

The main limitation of our assay is the lack of controllable and measurable shear stress between the vesicles and the pathogen, making it difficult to test the strength of the adhesion. It would be possible to immobilize the vesicles (for example by capturing them using a specific antibody) and run an adhesion assay under controlled flow conditions. However, in most cases, the *in vivo* shear stress between these epithelium-adhering pathogens and the membrane they adhere to is quite low [23], [24]. Another constraint of our assay is that the pathogen being investigated must be detectable by flow cytometry. This implies that its size must be between 1 and 150 µm and that, in the case of intracellular pathogens, infected cells must be distinguishable from non-infected cells. Epithelium-binding pathogens comprising mainly bacteria or parasites, fall mostly within this size range and their membrane could be easily stained with non-specific lipid dyes or DNA stains as used in the assay here. Interactions between viruses and epithelia may be more challenging to study.

### Some other potential uses of the assay

As demonstrated here in the application of the assay to placental malaria, one or more of the three components of the assay can be altered: vesicles, pathogen and co-incubation medium. For example, by cleaving specific receptors from the surface of the vesicles using an enzymatic treatment, it can be determined whether individual receptors are involved in adhesion and, if so, to what extent. One could also compare adhesion profiles of pathogens presenting different phenotypes and discover which one best correlates with adhesion. The co-incubation medium can also be altered to test the impact of exogenous agents on adhesion characteristics. Finally, being a flow-cytometry based assay, sorting of either pathogens and/or vesicles based on adhesion characteristics is feasible. For example, comparing the transcriptomes and surface molecule expression profiles of pathogens that do or do not adhere to vesicles could lead to the identification of pathogen molecules involved in adhesion. Also, epithelial membranes could be selected and isolated prior to the assay based on the expression of specific membrane markers. This is particularly useful when the epithelium is composed of various cell types such as the intestinal epithelium. For example, it has been shown that *Shigella flexneri*, a gram-negative bacteria causing diarrhea in humans, only adheres to M cells but there is no good model for these cells. Selecting membranes from M cells (based on their reactivity with mAb NKM 16–2–4 [25]) would allow, for the first time, to address the adhesion of *S. flexneri* specifically to the plasma membrane of these cells.

### Meaning of the new data generated on *Plasmodium falciparum*

By applying our assay to placental adhesion of *Plasmodium falciparum*-infected erythrocytes, we have validated our approach but also generated novel and important data.

#### Inter-vesicle variations in adhesion levels

Various vesicle preparations showed a wide variation in their capacity to support the adhesion of CSA-binding IE. Given the heterogeneity of the placental transcriptomes across anatomical sites [26], [27], vesicles prepared from various placental regions could support IE adhesion at different levels. However, given that villous tissue used to obtain each vesicle preparation was sampled from many sites in the placenta representative of the whole organ, the low inter-experiment variability of our assay (7%), and the comparable density, purity and mean fluorescence intensity of vesicle preparations, this variation in adhesion levels is likely to be due to qualitative differences between vesicle preparations and implies some degree of donor/biological variation, which could be an important predisposing factor to disease. Investigating further the bases of this donor variation would improve our understanding of placental adhesion and may lead to the identification of novel placental receptors or parasite variants with differential adhesion capacity. Adhesion percentages varied greatly across vesicle preparations but adhesion intensity was similar, suggesting that the difference between vesicle preparations may lie in the avidity of these vesicles for IE. Differential expression of CSA across vesicle preparations may explain the range of adhesion percentages observed. However, this would not account for the lack of hierarchy amongst vesicles when tested against different placental-type parasite lines (Figure 4b) unless the various parasite lines tested adhere to vesicles through different mechanisms.

#### Number of vesicles/IE

Considering the relatively small average diameter of the vesicles (±150 nm) compared to the infected erythrocyte (±7 µm), the adhesion of only 2–4 vesicles/IE cannot be explained by steric hindrance. In a similar assay examining adhesion of platelet microparticles to IE, similar numbers of platelet microparticles bound to IE were observed [28]. This low number of vesicles/IE could occur if the placental receptor is not uniformly expressed at the apical membrane, so that only a proportion of vesicles would express a placental receptor that can bind to IE and vesicles would therefore be limiting. This is unlikely given that the average number of vesicles/IE did not dramatically change with increasing vesicle/IE ratios, whereas adhesion levels did (Figure 2). Instead, we hypothesize that the knobs, these electron-dense molecular structures in which most IE adhesins reside, are not identical and that differences in the maturity and/or composition of knobs, as recently described [29], may explain low numbers of vesicles binding. The result of these variations may be that relatively few knobs can actually mediate placental adhesion.

#### Adhesion of var2csaKO IEs

BeWo-selected *var2csa*KO IE adhere to placental vesicles at the same level as the wild type placental-type line. This suggests that parasite molecules, other than VAR2CSA, can mediate high levels of placental adhesion. It is important to note that these IE do not consistently show the sex-specific, parity-dependent pattern of recognition by maternal antibodies typical of pregnancy-type parasites which could mean that such IE have a limited role in the pathogenesis of placental malaria. However, if one assumes that VAR2CSA is the default adhesin used by IE to adhere to the placenta and that adhesion-blocking immunity to VAR2CSA only develops after 2–3 successful pregnancies, alternative adhesins would only be expressed and engaged by parasites found in multigravidae and one would not be able to detect a parity-dependent recognition of these *var2csa*KO IE by maternal sera. The identification of the placental receptors supporting adhesion of these *var2csa*KO IE to vesicles would involve comparing the molecular profiles of vesicles that adhered or not to such IE. This is important in the context of placental malaria vaccine design.

A better understanding of the mechanisms underlying the pathogenetic process of epithelial adhesion of pathogens is needed. By its relevance, both in terms of adhesion matrix and pathogen, our innovative assay is powerful at defining key molecular epithelium-pathogen interactions. Being quantitative and high-throughput, it will be a valuable tool for the design and validation of adhesion-blocking strategies. With the capacity to be used in fundamental research as well as in drug discovery, and to be applied to various pathogens and epithelia with minimal or no modifications, our innovative assay is likely to assist a large number of teams in their research in host-pathogen interactions.

## Methods

### Purification of apical epithelial vesicles

Placentas were obtained after written informed consent from participants delivering by elective caesarian section at the Royal Women's Hospital, Melbourne. The study was approved by the Human Research Ethics Committee of the Royal Women's Hospital (project 08/33). All participants had uncomplicated pregnancies and delivered healthy singletons at term. Vesicles of the syncytiotrophoblast microvillous membrane were prepared within 30 minutes of delivery as described previously [14]. Briefly, the microvillous plasma membrane is sheared off the surface of the villi by isotonic tissue homogenization. Non-microvillous membranes are then aggregated by Mg^2+^ precipitation, allowing the isolation of the microvillous plasma membrane by differential centrifugation. Purified microvillous plasma membrane is then vesiculated by repeated passages through a 25G needle. Vesicles are stored at -80°C. The purity of the vesicle preparations was determined by the enrichment of alkaline phosphatase (AP, a specific marker of the microvillous membrane) compared to the starting placental homogenate [14]. Briefly, AP activity in placental homogenate and vesicles was quantified by the rate of conversion of p-nitrophenylphosphate at pH 9.8 over 2 minutes. Results were normalized to the protein concentration for each sample (determined by Lowry assay). Enrichment factor in AP was calculated as the ratio of AP activity in vesicles to initial placental homogenate. Before use in adhesion assays, vesicle preparation density was determined by flow cytometry acquisition against a reference fluorescent beads suspension of known density.

### *Plasmodium falciparum* culture

Malaria parasite lines were cultured in blood group O^+^ human erythrocytes, supplied by the Australian Red Cross Blood Service, synchronized and selected as described previously [19]. Parasite lines used were E8B (a non-placental type line) and the placental type lines CS2 (derived from E8B by selection on CSA), Pf2004-CSA, HB3-CSA and XIE-CSA (each panned on CSA to give high levels of CSA adhesion). A *var2csa*KO was previously generated on a CS2 background and was selected to bind to the trophoblast cell line BeWo [19].

### Adhesion assay

Vesicles were stained by PKH67 (Sigma), a fluorescent lipid dye, according to supplier's instructions and incubated for 1 h under gentle agitation with IE at 1% haematocrit and at least 5% parasitemia in adhesion medium (RPMI 1640–N-2-hydroxyethylpiperazine-N′-2-ethane sulphonic acid (HEPES) medium with 0.2% w/v NaHCO_3_ and 10% pooled heat-inactivated non-immune human serum, pH 7.2) in a final volume of 150 µL in 96-well U-bottom newborn calf serum-coated culture plates. Ethidium bromide (100 µg/mL final) was added for 15 minutes to stain parasites' DNA before washing off unbound vesicles by 3 centrifugation steps at 500 g for 3 minutes. After fixation in 0.5% formaldehyde, tubes were acquired using a FACSCalibur. A minimum of 10,000 IEs (EtBr^high^ erythrocytes) was acquired. A PKH67 fluorescence threshold was set using a negative control (IE without vesicles) so that 1% of IE was considered PKH67-positive. The readouts were the percentage of IE bound to vesicles among IE (percentage of adhesion) and the mean PKH67 fluorescence intensity (MFI) of IE (adhesion intensity).

Adhesion of IE to BeWo cells was quantified as described previously [19].

For adhesion-inhibition assays, IE were incubated with participants' sera (final dilution 1/10, 0.5% haematocrit) for 30 minutes at room temperature with constant agitation before performing the adhesion assay. In order to test the importance of VAR2CSA/CSA interaction in IE adhesion to vesicles, IE were incubated with 100 µg/mL of soluble CSA form bovine trachea (Sigma C9819) for 30 minutes at room temperature with constant agitation or vesicles were treated with chondroitinase-ABC (Sigma C3667; 0.1 IU/mL final) for 30 minutes in PBS with 0.1% bovine serum albumin.

### Immunofluorescence and scanning electron microscopy

After an adhesion assay, IE were smeared onto a microscope slide, air-dried and fixed in ice-cold methanol for 5 minutes. After re-hydration in PBS, vesicles were detected in a two-step immunofluorescence assay (1/5,000 monoclonal mouse anti-PLAP (Sigma-Aldrich A2951) followed by 1/1,000 AlexaFluor 488-conjugated rabbit anti-mouse IgG (Invitrogen A11059); both for 1 h at room temperature). Slides were mounted in ProLong Gold mounting medium with DAPI (Invitrogen). Images were acquired on a LSM Confocal Olympus FV1000 microscope. For scanning electron microscopy, cells were fixed post-assay for 15 minutes in 2.5% glutaraldehyde in 0.1 M sodium cacodylate pH 7.4, layered on a glass coverslip, washed twice with water, air dried, coated with platinum/palladium with a sputter coater (Polaron SC7640) and imaged using La Trobe University Electron Microscopy Facility's field emission scanning electron microscope (FESEM, Jeol JSM 6340F) operating at 5 keV.

### Statistical analysis

Mean and standard errors to the mean were compared by ANOVA using Stata software (version 10). P≤0.05 denoted statistical significance.
